# Antiplatelet and Antithrombotic Activities of *Lespedeza cuneata* via Pharmacological Inhibition of Integrin *α*IIb*β*3, MAPK, and PI3K/AKT Pathways and FeCl3-Induced Murine Thrombosis

**DOI:** 10.1155/2024/9927160

**Published:** 2024-02-09

**Authors:** Abdul Wahab Akram, Evelyn Saba, Man Hee Rhee

**Affiliations:** ^1^Department of Veterinary Medicine, College of Veterinary Medicine, Kyungpook National University, Daegu 41566, Republic of Korea; ^2^Department of Veterinary Biomedical Sciences, Faculty of Veterinary and Animal Sciences, Pir Mehr Ali Shah Arid Agriculture University, Rawalpindi 46000, Pakistan; ^3^Companion Animal Medical Institute, College of Veterinary Medicine, Kyungpook National University, Daegu 41566, Republic of Korea

## Abstract

Cardiovascular diseases (CVDs) have been the major cause of mortality all around the globe. *Lespedeza cuneata* abbreviated as *L. cuneata* with the authority name of Dumont de Courset (G. Don) is a perennial flowering plant commonly grown in Asian countries such as Korea, Japan, China, and Taiwan. We aimed to investigate the *L. cuneata* extract's antiplatelet and antithrombotic properties as GC-MS analysis indicated that the extract contained short-chain fatty acids, which have been reported to possess beneficial cardiovascular effects. *L. cuneata* was extracted using water, 50% EtOH, 70% EtOH, and 100% EtOH. For *in vitro* antiplatelet analysis, washed platelets were prepared and incubated with *L. cuneata* with 200 *μ*g/mL of 50% EtOH in the presence of 1 mM of CaCl_2_ for 1 minute followed by agonist (collagen 2.5 *μ*g/mL or ADP 10 *μ*M or thrombin 0.1 U/mL) stimulation for 5 minutes over light transmission aggregometer. Scanning electron microscopy was performed to assess platelet shape change. ATP release and intracellular calcium mobilization were quantified to assess the granular content. Fibrinogen-binding assay and clot retraction assay assessed integrin *α*IIb*β*3-mediated inside-out and outside-in signaling. Protein phosphorylation expression was investigated by western blot analysis. Finally, the *in vivo* antithrombotic efficacy was investigated by oral dosage of *L. cuneata* 200 and 400 mg/kg and aspirin 100 mg/kg for 7 days, and tail bleeding and FeCl_3_-induced murine thrombus model were performed. *In vitro* platelet aggregation and platelet shape change were dose-dependently suppressed by *L. cuneata*. Calcium mobilization, dense granules secretion, integrin *α*IIb*β*3-mediated inside-out and outside-in signaling, and protein phosphorylation of MAPK and PI3K/Akt pathways were significantly inhibited. *In vivo* assays revealed that *L. cuneata* prevents side effects of synthetic drugs via nonsignificantly increasing bleeding time and improving coronary artery blood flow and animal survival. Our results demonstrate that *L. cuneata* exhibited potent antiplatelet and antithrombotic effects and can be considered a potential herbal medicine with cardioprotective effects.

## 1. Introduction

Cardiovascular diseases (CVDs) have affected the majority of population worldwide [1]; among them, coronary heart disease is the most common type of heart disease, causing mortality of around 375,476 people in 2021. According to the heart disease and stroke statistics update of 2021, heart diseases are responsible for 1 in 4 fatalities in the United States [2]. About 1 in 20 adults aged 20 and older have CVD (about 5%) [3]. Statistics from China in 2023 reported that two out of every five deaths are due to CVD [4]. Over the past 50 years, CVD mortality has declined, but cardiovascular events such as heart attacks and strokes remain by far the leading cause of death in the European Union, accounting for 36% of all deaths and impacting the lives of 60 million people who live with CVD. More broadly, CVD accounts for 47% and 39% of all deaths in women and men, respectively, in the wider European Regions [5]. Although the total mortality rate of cardiovascular disease (CVD) in South Korea has significantly decreased, heart disease remains the second leading cause of death, and ischemic heart disease (IHD) mortality has continuously increased until recently [6].

The pathophysiology of CVDs involves several factors; among them, hyperactive platelets are the main offenders, leading to platelet plug formation and vascular stenosis, which may result in ischemic stroke [7]. Originating from megakaryocytes, platelets express various receptors, adhesion molecules on their surface, and granules in the inner compartments, which contain downstream effector molecules [8]. Glycoprotein Ib/V/IX interacts with von Willebrand factor after vascular damage, resulting in platelet receptors GPVI and integrin *α*IIb*β*3 binding to collagen, causing outside-in signaling, platelet activation, phosphatidyl serine exposure, and release of granule contents [9]. Subsequent thrombin generation, platelet binding to integrin *α*IIb*β*3, and stable platelet thrombus fasten the injured site and make a basis to heal the damaged vascular site. Moreover, platelets contain mitogen-activated protein kinases (MAPKs), such as ERK1/2 and JNK1, which are involved in apoptosis, migration, and proliferation. Many agonists, including collagen, ADP, and thrombin, phosphorylate MAPKs, which are crucial for both “inside-out” and “outside-in” signaling [10]. In addition, essential for platelet activation and aggregation is the PI3K/Akt signaling pathway. Moreover, tyrosine phosphorylation-based signaling pathways triggered by GPVI or *α*IIb*β*3 require PI3Ks [11].

The majority of the countries have a long history of employing medicinal plants for diagnosis, treatment, and maintenance of personal hygiene as well as for use as food supplements, cosmetics, and scents. The world's medicinal and aromatic plant diversity is highest in the Asia-Pacific region. Global demand for herbal medicine has seen a tremendous increase as evident from a report released by the Research and Information System (RIS) for Developing Countries. According to this report, the herbal sector is expected to reach a global market size of US$746.9 billion in 2022, compared to a projected US$657.5 billion in 2020 [12]. Meanwhile, synthetic drugs have been successfully used to treat and prevent CVDs, but no cures are without serious side effects. For instance, clopidogrel occasionally causes aplastic anemia and thrombocytopenic purpura, and the most commonly used antiplatelet drug aspirin sometimes causes severe stomach ulcers or persistent bleeding with high levels of resistance to these drugs [13]. Considering this, the ethnomedical approach could be a promising strategy for preventing CVDs and their complications [14]. Natural herbal components and the Mediterranean diet contain bioactive compounds that can modulate platelet activity. These natural approaches may help lower the risk of thrombosis [15].

*Lespedeza cuneata* G. Don belongs to the family Fabaceae and the genus Leguminosae. *L. cuneata* is a flowering plant commonly grown in Asian countries and has been reported for its antioxidative and anti-inflammatory effects [16], scalp-improving properties [17], prostatic hyperplasia [18], early atherosclerosis [19], and whitening skin [20]. However, its pharmacological action in preventing platelet aggregation and antithrombotic properties has not been explored yet. Therefore, this study aimed to explore the pharmacological action of *L*. *cuneata* on attenuating platelet aggregation via *in vitro* and *in vivo* models. To the best of our knowledge, this will be the first study to unravel antiplatelet and antithrombotic properties of *L. cuneata* via inhibition of integrin *α*IIb*β*3, MAPK, PI3K/AKT pathways, and FeCl_3_-induced murine thrombosis model.

## 2. Materials and Methods

### 2.1. Reagents

Collagen, ADP, and thrombin were obtained from Chrono-Log Co. (Collagen cat # 385 ADP cat #384 Thrombin cat #386, CHRON-LOG Corporation, Havertown, PA, USA). Paraformaldehyde (CAS no. 30525-89-4) and glutaraldehyde (CAS no. 111-30-8) were purchased from Sigma-Aldrich (St. Louis, MO, USA). The ATP Assay kit was obtained from the Biomedical Research Service (REF. 373, BMR, 3434 Main St, Buffalo, NY, USA). Thrombin from human plasma was purchased from Sigma-Aldrich (Lot # SLCF9776 CAS no. 9002-04-4 647-014-00-9). Fura 2-AM (2-acetoxymethyl) (CAS number: 108964-32-5) and Alexa Fluor 488-conjugated fibrinogen (Cat # A32723) were obtained from Invitrogen (Eugene, OR, USA). All antibodies were supplied by Cell Signaling (Beverly, MA, USA).

### 2.2. Extraction and Procurement of Plant Samples

Plant samples were extracted as described previously [21]. In brief, *L. cuneata* was extracted for 2 h with distilled water and 50%, 70%, and 100% EtOH at 100°C for water and 80°C for EtOH in a 1 : 20 ratio (plant: solvent w/v). After extraction, these samples were filtered using filter paper (Whatmann™ no.4). Then, the samples were evaporated via rota vapor and placed at −70°C overnight to freeze. The samples were then freeze-dried for 3-4 days at −55°C to obtain a powdered form. Ultrapure water was used to dissolve the water extract and dimethyl sulfoxide (DMSO) for ethanol extracts in particular concentrations for the evaluation of the samples. The characterization of the extract was done with GC-MS as explained in the following.

### 2.3. GC-MS Analysis

**Figure 1:**
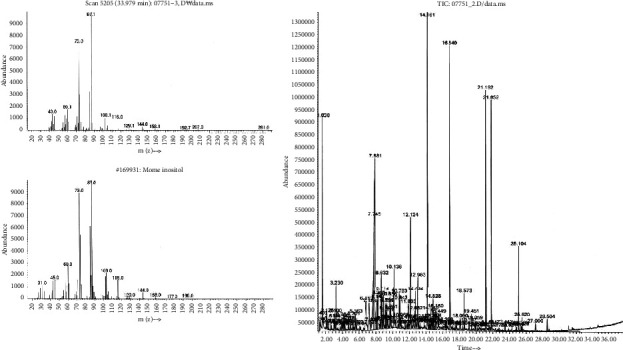
Gas chromatography-mass spectrometry analysis. To separate and measure *Lespedeza cuneata* compounds, the GC-MS instrument was outfitted with a 30 m × 0.25 mm (i.d. DB-5MS) chromatography column and an Agilent 5975C mass selective detector. At 250°C, the extract was injected. The source and transfer lines had temperatures of 230°C and 280°C, respectively. The column temperature was initially set at 70°C for 1 min and then increased to 300°C at a rate of 5°C/min, remaining at this ultimate temperature for 30 min. Scan and electron ionization modes were used to obtain mass spectrometry data.

The GC-MS analysis was performed using an Agilent 7890A GC instrument (Agilent Technologies, Santa Clara, CA, USA). The instrument was equipped with a 30 m × 0.25 mm (i.d. DB-5MS) chromatography column and an Agilent 5975C mass selective detector. The extract was injected at a temperature of 250°C. The source and transfer line temperatures were set at 230°C and 280°C, respectively. The column temperature was initially set at 70°C for 1 min and then increased at a rate of 5°C/min to a final temperature of 300°C, which was maintained for 30 min. MS data were obtained in scan and electron ionization modes to analyze the *L. cuneata* compounds.  Figure 1 and Table 1 represent the peaks and composition of compounds present in the *L. cuneate* extract.

### 2.4. Experimental Animals

Male Sprague–Dawley (SD) rats, 7 weeks old and weighing 240–260 g, C57BL/6J male mice, weighing 20–22 g, and ICR mice, weighing 30–40 g, were acclimated to an environment control room maintained at approximately 23 ± 2°C and 50% ± 10% humidity, with a 12-h light/dark cycle. Male SD rats were used to obtain sufficient blood for platelet isolation for *in vitro* experiments, whereas C57BL/6J and ICR mice were used for *in vivo* experiments.

All animal care and experimental procedures were carried out in strict accordance with internationally accepted guidelines on the use of laboratory animals and the protocols were approved by the Animal Care Committee of the College of Veterinary Medicine, Kyungpook National University, Daegu, South Korea (permit no. KNU-2015-60).

### 2.5. Preparation of Washed Rat Platelets

To prepare washed platelets, SD rat blood was obtained. Blood was drawn by cardiac puncture with a syringe containing the anticoagulant solution acid citrate dextrose (ACD) and then transferred to a round bottom tube containing Tyrode's buffer and ACD in a 1 : 4 ratios under light anesthesia with diethyl ether. To obtain platelets from the whole blood, initially, it was centrifugated at 170 × *g* for 7 min and then another round of centrifugation at 350 × *g* for 10 min to get washed platelets. For platelet aggregometry analysis, the collected platelets were balanced to 3 × 10^8^ cells/mL by adding Tyrode's buffer.

### 2.6. Platelet Aggregation Assay and SEM Analysis

As reported previously [22], light transmission aggregometry was performed to access platelet aggregation and inhibition by the plant sample. In brief, washed platelets were obtained from rat blood and incubated for 1 min with 1 mM calcium chloride and varying concentrations of *L. cuneata* (50 *μ*g/mL, 100 *μ*g/mL, and 200 *μ*g/mL) or DMSO. After 1 min, collagen, ADP, and thrombin (collagen 2.5 *μ*g/mL or ADP 10 *μ*M or thrombin 0.1 U/mL) were added to stimulate platelets and aggregation was stopped after 6 min. Light transmission through the glass tube was read as percent transmission by the light transmission aggregometer.

For SEM, after incubation for 5 min with *L. cuneata* and agonists, 0.5% paraformaldehyde and 0.5% osmium tetroxide were used for platelet fixation, and platelets were then dehydrated with increasing concentrations of EtOH from 50% to 100%, followed by freeze-drying at −55°C. Platelet shape change was assessed by ultrastructure pictures captured using a field transmission electron microscope (SU8220; Hitachi, Japan).

### 2.7. [Ca^2+^]_i_ Mobilization

Fura-2/AM at a concentration of 5 M is incubated with platelet-rich plasma (PRP) for 1 h at 37°C. Fura-2-loaded platelets, at a concentration of 3 × 10^8^/mL, were then stimulated with collagen for 5 min after preincubation with a plant sample in the presence of 1 mM CaCl_2_. Fura-2 fluorescence in the cytosol is calculated using the formula [Ca^2+^]_i_ = 224 nM (*F F*_min_)/(*F*_max_* F*), where *F* is the dissociation constant and *F*_min_ and *F*_max_ are the fluorescence intensities.

### 2.8. ATP Release Assay

Collagen was used to stimulate washed platelets after they have been preincubated with various doses of plant extract for 5 min at 37°C. After the aggregation reaction, the platelet mixture was centrifuged at 12000 rpm to extract the supernatant, and the amount of ATP secreted (in the supernatant) was then determined using an ATP Assay kit and a luminometer.

### 2.9. Fibrinogen-Binding Assay

Washed platelets were pretreated with *L. cuneata* and an antifibrinogen antibody. Then, paraformaldehyde 0.5% was used to fix the platelets. A flow cytometer (FACS Aria III) was used in the cytometric analysis.

### 2.10. Clot Retraction

By evaluating clot retraction as previously described [23], after incubating PRP (250 L) with the vehicle, *L. cuneata*, or Y27632 (Rock inhibitor) for 2 min, the volume was increased to 1 mL by adding red blood cells (RBCs, 5 L) and Tyrode's buffer. The injection of thrombin (1 U/mL) caused the clot to retract, which was then monitored for 90 min at room temperature. To evaluate clot retraction, clot weight was lastly assessed.

### 2.11. Western Blotting

Various quantities of *L. cuneata* were preincubated with washed platelets for 1 min and stimulated with collagen for 5 min. Platelet aggregation was stopped by adding a lysis buffer (PRO-PREP; iNtRON Biotechnology, Seoul, Korea), and protein concentration was determined using the BCS assay (PRO-MEASURE; iNtRON Biotechnology). Total platelet proteins were isolated, separated by 10% sodium dodecyl-sulfate polyacrylamide gel electrophoresis and then transferred to polyvinylidene difluoride (PVDF) membranes. The membranes were blocked with 5% skim milk, probed with appropriate antibodies (phospho-ERK, phospho-JNK, phospho-p38MAPK, phospho-PI3K, phospho-Akt, etc.), and visualized using enhanced chemiluminescence.

### 2.12. *In Vivo* Tail Bleeding and the FeCl_3_-Induced Thrombus Model

To evaluate tail bleeding assay and FeCl_3_-induced thrombus formation, four treatment groups were treated orally for 7 days (group 1 = saline, group 2 = ASA, and group 3 and 4 = low and high dose of the 50% EtOH extract of *L. cuneata*) using C57BL/6J and ICR mice, respectively. A tail bleeding assay was carried out as previously reported 1 h after the last treatment while the FeCl_3_-induced thrombus formation assay was carried out 20 min after the last treatment as previously reported [24, 25]. C57BL/6J mice have been established for the tail bleeding assay [22] but here, we used ICR mice for the FeCl_3_-induced thrombus model as Shim et al. recently reported that ICR mice showed better dose responses in thrombus formation and stability compared to the C57BL/6N mice [26].

### 2.13. Statistical Analysis

Acquired data were subjected to one-way analysis of variance and post hoc Dunnett's test (SAS Institute Inc., Cary, NC, USA) to determine the statistical significance of the observed differences. The provided information is displayed as the mean ± standard deviation (SD). Statistical significance was defined as a *p* value of 0.05 or lower.

## 3. Results

### 3.1. Gas Chromatograph-Mass Spectrometry (GC-MS) Analysis

**Figure 2:**
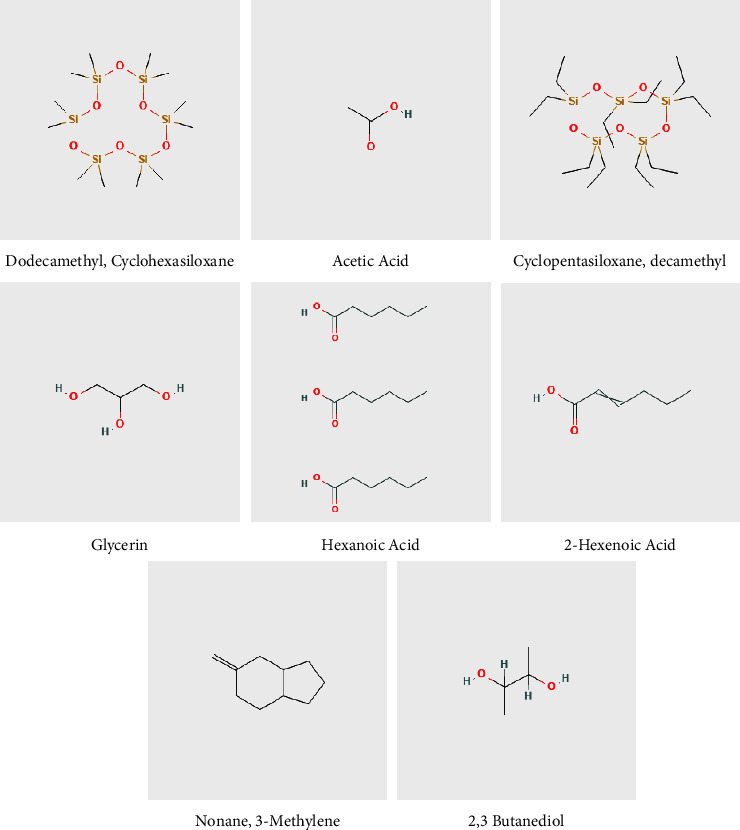
Chemical structures of the single compounds present in the *Lespedeza cuneata*.

When comparing the peaks obtained at different melting points to standard chemical analysis library data, GC-MS analysis demonstrated the structural similarity of *L. cuneata* to inositol (Figure 1). However, active ingredients found in our sample with the highest retention time were as follows: 3-hexenoic acid, 3-acetyl-4-methyl-3-pyrrolin-2-one, cyclohexasiloxane, dodecamethyl, hex-3-enoic acid, 2(4H)-benzofuranone,5,6,7 7a-tetrahydro-4,4, 7a trimethyl, 3H-pyrazol-3-one, 2,4-dihydro-5-(3-nitrophenyl)-2-phenyl, acetic acid, cyclopentasiloxane, decamethyl, and glycerin (Table 1). The structure of active compounds present in the extract is shown in Figure 2.

## 4. Results for the *In Vitro* Study

### 4.1. *L. cuneata* Inhibits Agonist-Induced Platelet Aggregation

**Figure 3:**
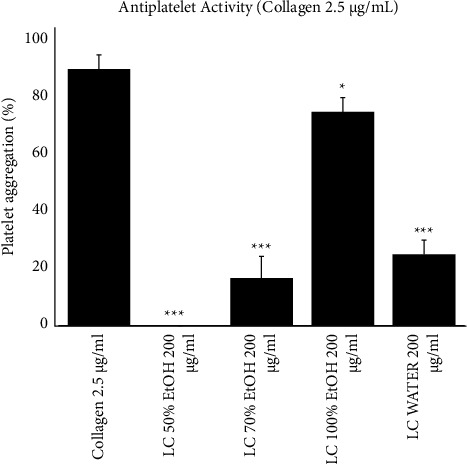
Antiplatelet activity of *Lespedeza cuneat*a. Washed platelets were incubated for 1 min with varying concentrations of *L. cuneata* with 50%, 70%, and 100% EtOH and water extracts or vehicle (DMSO) along with 1 mM calcium chloride, stimulated with collagen for 5 min. The graph represents the mean ± SEM of at least three independent experiments. ^*∗*^*p* < 0.05, ^*∗∗*^*p* < 0.01, and ^*∗∗∗*^*p* < 0.001 compared with the agonist-treated group.

After extracting plant samples using different solvents such as 50% EtOH, 70% EtOH, 100% EtOH, and water, antiplatelet effects by inhibiting platelet aggregate formation during light transmission aggregometry was determined. Among them, 50% EtOH showed the highest platelet aggregation inhibition at 200 *μ*g/mL (Figure 3). Therefore, *L. cuneata* with 50% EtOH was selected for further evaluation for its mechanistic pathways to present antiplatelet and antithrombotic activities.

### 4.2. *L. cuneata* Inhibits Agonist-Induced Changes in the Platelet Shape

**Figure 4:**
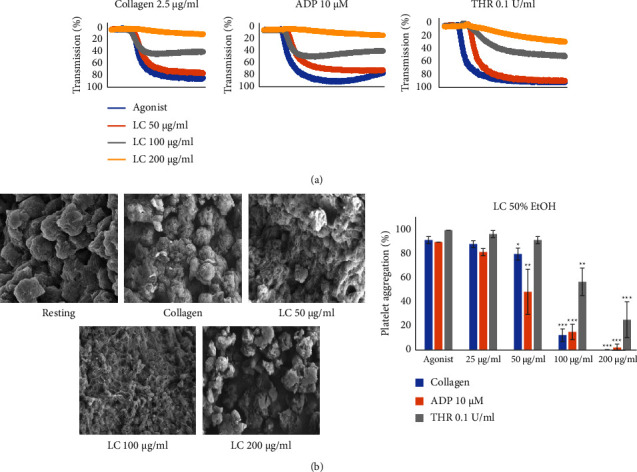
Effects of *L. cuneata* on agonist-induced platelet aggregation and changes in the platelet shape. (a) Agonists, such as collagen, ADP, and thrombin, were preincubated with washed platelets for 1 min in a light transmission aggregometer in the presence of 1 mM calcium chloride (CaCl_2_). This has been followed by stimulation for 5 min with constant stirring at 37°C using various agonists. ^*∗*^*p* < 0.05, ^*∗∗*^*p* < 0.01, and ^*∗∗∗*^*p* < 0.001 compared with agonist. (b) The shape change and aggregation of platelets were evaluated using scanning electron microscopy.

Initial screening demonstrated the effectiveness of *L. cuneata* with 50% EtOH against various agonists, i.e., collagen, ADP, and thrombin (collagen 2.5 *μ*g/mL or ADP 10 *μ*M or thrombin 0.1 U/mL) using light transmission aggregometry. *L. cuneata* substantially and dose-dependently inhibited agonist-induced platelet aggregation (Figure 4(a)), while *L. cuneata* dose-dependently suppressed agonist-induced platelet shape change, assessed using scanning electron microscopy (SEM) (Figure 4(b)).

### 4.3. *L. cuneata* Reduces [Ca^2+^]_i_ Mobilization and ATP Release from Alpha Granules

The amount of intracellular calcium release was quantified by the following:

(1) $$\begin{array}{c} {\left[{\text{Ca}}^{2+}\right]}_{i}=\frac{224\text{nM}\left(FF_{\min}\right)}{\left(F_{\max}\;F\right)}. \end{array}$$

**Figure 5:**
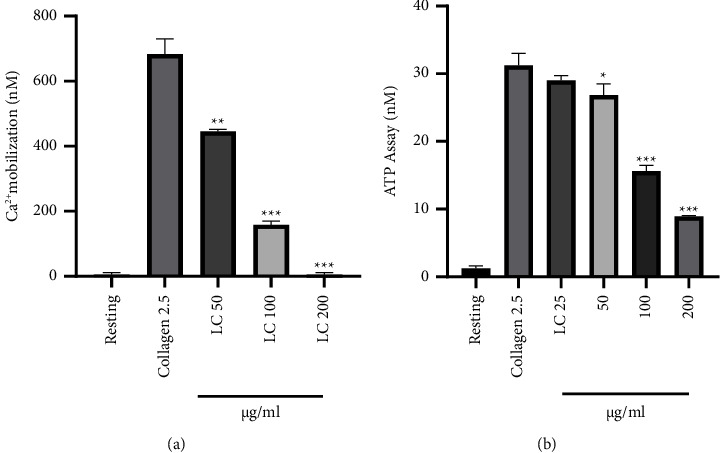
*L. cuneata* inhibits collagen-stimulated platelet intracellular calcium ion ([Ca^2+^]_i_) mobilization and ATP release from alpha granules. (a) The amount of intracellular calcium release was quantified by using this formula, [Ca^2+^]_i_ = 224 nM (*F F*_min_)/(*F*_max_* F*), where *F*_min_ and *F*_max_ are the fluorescence intensities at extremely low and very high Ca^2+^ concentrations, respectively, and *F* is the dissociation constant of the Fura-2-Ca^2+^complex. (b) ATP release in collagen-stimulated rat platelets was measured using supernatant from the aggregation reaction using an ATP ELISA kit. ^*∗*^*p* < 0.05, ^*∗∗*^*p* < 0.01, and ^*∗∗∗*^*p* < 0.001 compared with the agonist.

Here, *F*_min_ and *F*_max_ are the fluorescence intensities at extremely low and very high Ca^2+^ concentrations, respectively, and *F* is the dissociation constant of the Fura-2-Ca^2+^ complex. Our results demonstrated that the dose regimen for *L. cuneata* significantly and dose-dependently inhibited calcium mobilization, whereas 200 *μ*g/mL completely abolished the increase in calcium concentration induced by collagen stimulation (Figure 5(a)).

ATP release was measured using supernatant from the aggregation reaction using an ATP ELISA kit. Our results demonstrated that 50, 100, and 200 *μ*g/mL dose-dependently inhibited ATP release (Figure 5(b)).

### 4.4. *L. cuneata* Reduced Fibrinogen Release from *δ*-Granule Secretion and Attenuated Inside-Out and Outside-In Signaling

**Figure 6:**
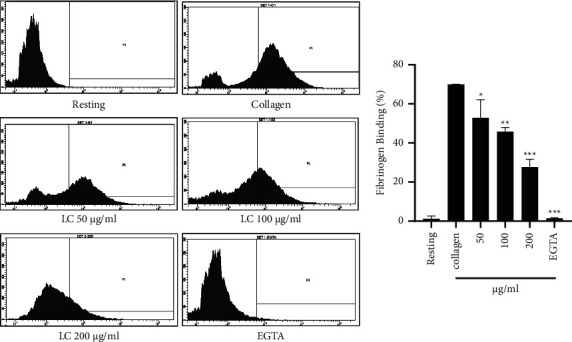
Assessment of integrin *α*IIb*β*3-mediated inside-out signaling in the presence of *Lespedeza cuneata*. In the presence of an antifibrinogen antibody, washed platelets were pretreated with the plant extract and activated with an agonist for 5 min. After that, paraformaldehyde 0.5% was used to fix the platelets. A flow cytometer (FACS Aria III) was used to assess integrin *α*IIb*β*3 activation which was significantly induced by collagen and reduced dose dependently by the dose of treatment. ^*∗*^*p* < 0.05, ^*∗∗*^*p* < 0.01, and ^*∗∗∗*^*p* < 0.001 compared with the agonist-treated group.

**Figure 7:**
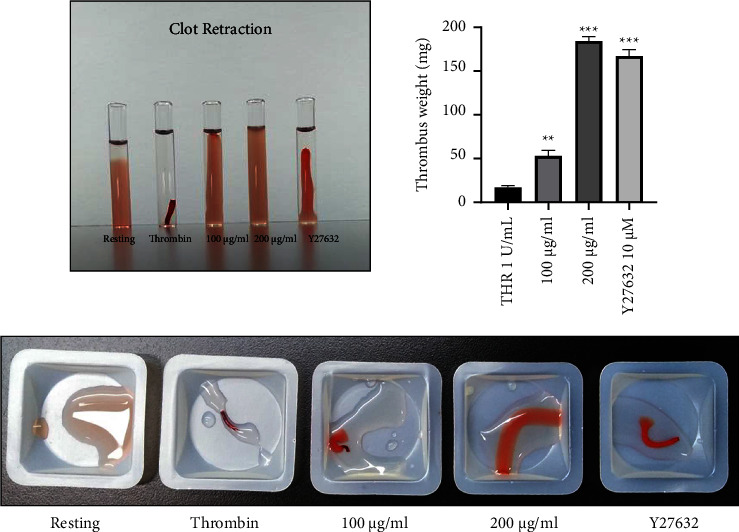
Assessment of clot retraction by outside-in signaling in the presence of *Lespedeza cuneata*. After incubating PRP (250 L) with the vehicle, *L. cuneata*, or Y27632 (rock inhibitor) for 2 min, the volume was increased to 1 mL by adding RBCs (5 L) and Tyrode's buffer. The injection of thrombin (1 U/mL) caused the clot to retract, which was then monitored for 90 min at room temperature. To evaluate clot retraction, clot weight was lastly assessed. ^*∗∗*^*p* < 0.01 and ^*∗∗∗*^*p* < 0.001 vs. thrombin (THR).

The effects of the *L. cuneata* extract at various doses on collagen-induced platelet signaling were investigated. Our results revealed that *L. cuneata* reduced fibrinogen interaction to integrin *α*IIb*β*3 significantly to inhibit inside-out signaling (Figure 6). In addition, the *L. cuneata* with 50% EtOH extract significantly and dose-dependently decreased clot retraction and outside-in signaling to prevent platelet shape change (Figure 7).

### 4.5. *L. cuneata* Attenuates MAPK and PI3K/Akt Phosphorylation

**Figure 8:**
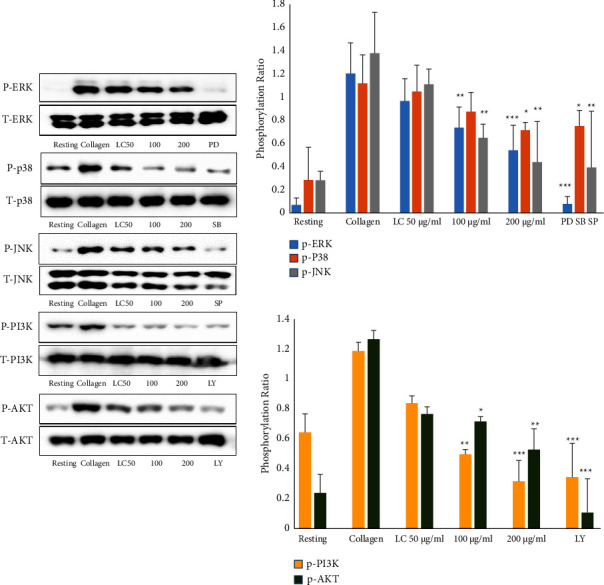
Western blot analysis to assess protein phosphorylation under *Lespedeza cuneata* stimulation. Various quantities of *L. cuneata* extracts with 1 mM CaCl_2_ were preincubated using washed platelets for 1 min at 37°C before collagen stimulation for 5 min with constant stirring. By adding a lysis buffer, platelet aggregation stopped, and the protein concentration was calculated using the BCS assay (PRO-MEASURE; iNtRON Biotechnology). In a 10% SDS-PAGE, total platelet proteins were isolated, and then they were transferred to PVDF membranes. Membranes were blocked with 5% skim milk, probed with the appropriate antibodies (phospho-ERK, phospho-JNK, phospho-p38MAPK, phospho-PI3K, phospho-Akt, etc.), and then observed using enhanced chemiluminescence. ^*∗*^*p* < 0.05, ^*∗∗*^*p* < 0.01, and ^*∗∗∗*^*p* < 0.001 compared with the collagen.

Protein phosphorylation during platelet aggregation inhibition was investigated using Western blot. The results demonstrated significant inhibition of the phosphorylation of MAPK and PI3K/AKT by treatment with different doses of *L. Cuneata* with 50% EtOH (Figure 8).

## 5. Result for the *In Vivo* Study

### 5.1. *L. cuneata* Prevents Thrombosis and Regulates Hemostasis

**Figure 9:**
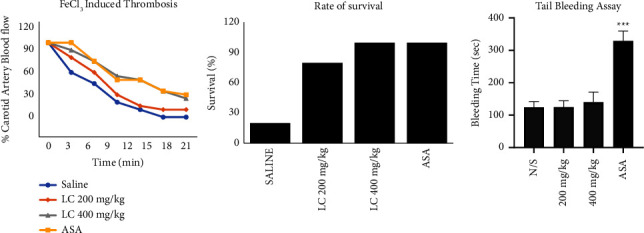
*L. cuneata* prevents thrombosis and regulates hemostasis. To evaluate FeCl_3_-induced thrombus formation and the tail bleeding assay, four treatment groups were treated orally for 7 days (saline, ASA, or a low and high dose of the 50% EtOH extract of *L. cuneata*) using C57BL/6J and ICR mice, respectively. Mice were anesthetized, and FeCl_3_-induced thrombus formation and the tail bleeding assay were carried out almost 1 h after the last treatment. ^*∗∗∗*^*p* < 0.001 compared with the agonist.

To evaluate the side effects caused by remedial substances on thrombosis and hemostasis, a FeCl_3_-induced thrombus model and a tail bleeding assay was performed using acetylsalicylic acid (ASA) as a positive control. After induction of thrombus with 35% FeCl_3_, *L. cuneata* not only improved the blood flow but also the survival rate of mice similar to the ASA group. On the other hand, *L. cuneata* does not possess the side effect of aspirin by preventing increased bleeding time (Figure 9).

## 6. Discussion

A myriad of ailments known as CVDs affects the heart and blood vessels. Cardiovascular conditions include thrombosis, acute myocardial infarcts, and coronary heart artery disease. Platelets have an important role in the treatment and prevention of CVDs and are currently the subject of numerous investigations. Antiplatelet medications lower death rates caused by myocardial infarction.

At areas of atherosclerotic plaque rupture, changes in the blood flow promote platelet activation and arterial thrombus formation [27]. Plant extracts having antiplatelet effects can be a better remedy for thrombotic and CVDs assessed by providing similar conditions to blood flow as in light transmission aggregometry. Intra-arterial coronary collateral vessels may contract because of ATP, and platelet activity in coronary arteries connected to the collateral vasculature may result in collateral vasoconstriction, limiting blood flow to the dependent myocardia [28]. The terminal stage of platelet aggregation can be recognized by inside-out signaling and the activation of integrin *α*IIb*β*3, whose activation is crucial during this process. In other words, integrin *α*IIb*β*3 connects itself to another integrin through a bridge network resembling fibrinogen. The blood clot is finally tightened and becomes stable [29].

Collagen, thrombin, and ADP by initiating subsequent signaling processes via the activation of the GPVI, PAR, and P2Y12 receptor signaling pathways cause significant platelet aggregation. We tested the antiplatelet activity of water, 50%, 70%, and 100% EtOH *L. cuneata* extracts. Figure 3 demonstrates that *L. cuneata* with 50% EtOH showed the greatest reduction in platelet activity induced by collagen. SEM images of the platelet shape revealed a change in morphology from discoid to a rounded shape containing filopodia upon activation with collagen which was prevented by treatment with *L. cuneata* with 50% EtOH (Figure 4) [30].

The alpha (a) granules in platelets are filled with sticky ligands including fibrinogen, fibronectin, P-selectin, and dense (d) granules such as Ca^2+^ and ATP. Following platelet activation, these granules are secreted, which improves platelet adherence, shape change, and aggregation [31]. By preventing Ca^2+^ release and fibrinogen binding, *L. cuneata* with 50% EtOH was found to block the secretion of both dense and alpha granules, lowering platelet activation, adhesion, shape change, and aggregation (Figure 5) as reported previously by Holmsen [32].

The ability of plant extracts to regulate platelet aggregation and activation can be determined by testing them for the inhibition of fibrinogen binding to *α*IIb*β*3 using flow cytometry [33]. Similarly, in our results, *α*IIb*β*3 integrins were activated by collagen and a reduction in *α*IIb*β*3 integrins was observed after *L. cuneata* treatment (Figure 6). Through cytoskeletal alterations in actin, GTPases control platelet adherence, shape change, and clot retraction. By phosphorylating the myosin light chain, Rho kinases are downstream regulators that affect the actin cytoskeleton in response to RhoA. Y-27632 has been employed to investigate the role of Rho kinase in promoting clot retraction [34]. Src kinase, a member of the SFKs family, plays a significant role in integrin *α*IIb*β*3-mediated signaling, which may be also implicated in clot retraction [11]. Abciximab and eptifibatide are *α*IIb*β*3 antagonists that were previously consumed for preventing occlusive vascular events in atherosclerosis [35]. Similar results were obtained when platelets were incubated with thrombin from human plasma or *L. cuneata* (Figure 7).

MAPKs, including ERK, JNK, and p38 MAPK, and their phosphorylation result in the release of granules, which increase platelet aggregation [36]. Furthermore, MAPK and PI3K/Akt play critical roles in platelet activation by influencing calcium mobilization, granule secretion, and platelet aggregation [37]. In our study, the plant extract inhibited these molecules and significantly reduced the phosphorylation of MAPK and PI3K/Akt, exhibiting a potential method of the inhibition of platelet activities (Figure 8).

Finally, the inhibitory effects of *L. cuneata* with 50% EtOH on thrombus formation and hemostasis were evaluated with a FeCl_3_-induced thrombus model and a tail bleeding assay. By inhibiting platelet activation, we found that treatment with *L. cuneata* with 50% EtOH significantly reduced thrombus formation and modestly increased bleeding duration compared with treatment with ASA control [21] (Figure 9).

We speculate that the potent antiplatelet and antithrombotic activity of the *L. cuneata* extract via GC-MS is due to the presence of single components similar to inositol, which has been reported to treat high-fat diet-induced cardiac dysfunction [38] and prevent vascular calcification [39]. Active components evaluated by GC-MS analysis in our study have previously been reported for their antiplatelet and antithrombotic activities such as acetic acid [40], glycerin [41], hexanoic acid, 2-hexanoic acid [42], 3-methylene [43], nonane [44], and 2,3 butanediol [45] (Figure 1 and Table 1).

**Figure 10:**
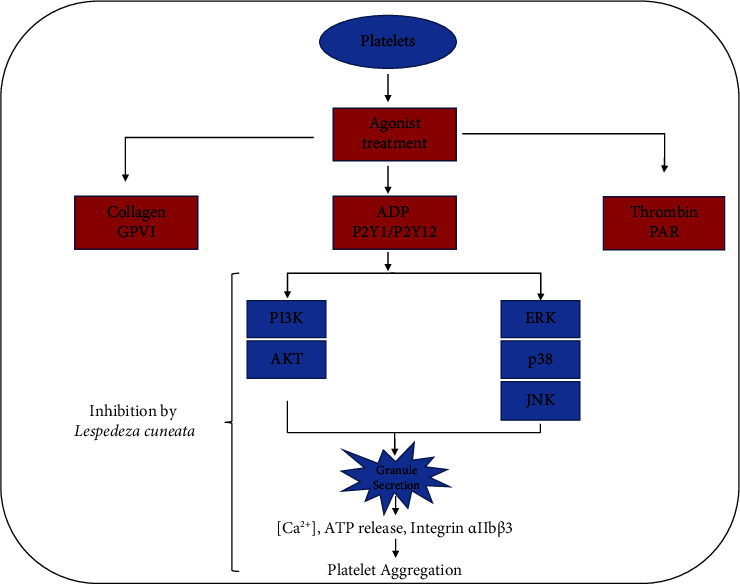
Inhibitory mechanism of *L. cuneata* on the platelet signaling pathway. *L. cuneata* inhibits the phosphorylation of MAPKs (ERK, JNK, and p38MAPK) and PI3K/Akt, limiting the release of granules and reducing platelet aggregation. In addition, by affecting calcium mobilization, granule secretion, and platelet aggregation, MAPK and PI3K/Akt, and downstream effectors of SFK, play crucial roles in platelet activation inhibition.

The results of our study are summarized in Figure 10, which show potent cardioprotective effects of *L. cuneata.*

## 7. Conclusion

This study used 50% EtOH extract, the most effective component, to test *L. cuneata* as herbal medication. As evidenced by our results *L. cuneata* dose dependently and significantly inhibited fibrinogen binding, alpha and dense granule release, protein expression, MAPK, and PI3K/Akt signaling pathways after agonist-induced platelet activation. Finally, *L. cuneata* did not have the negative consequences of prolonged bleeding times caused by the most commonly used antiplatelet medications (i.e., aspirin and clopidogrel). These results demonstrate that *L. cuneata* can be a potent herbal plant extract to substitute synthetic antiplatelet medications to prevent CVDs.

## Figures and Tables

**Table 1:** GC-MS analysis of major compounds present in the 50% EtOH *L. cuneata* extract.

Retention time (RT)	Chemical compounds	Area (%)
16.84	Dodecamethyl, cyclohexasiloxane	7.49
1.63	Acetic acid	4.05
12.12	Cyclopentasiloxane, decamethyl	3.31
6.81	Glycerin	2.74
7.18	Hexanoic acid	2.19
8.24	2-Hexenoic acid	2.05
10.13	Nonane, 3-methylene	1.87
3.23	2,3-Butanediol	0.99

## Data Availability

The data used to support the findings of this study are available on request from the corresponding author.

## Supplementary Materials

Supplementary Materials The full-length blots for the gel images are shown in the supplementary material. Supplementary figure S1a = P-ERK pathway in platelets. Supplementary figure S1b = P-p38 pathway in platelets. Supplementary figure S1c = P-JNK pathway in platelets. Supplementary figure S1d = P-Akt pathway in platelets. Supplementary figure S1e = PI3K pathway in platelets.

## References

[1] heartdisease (2013). The underlying cause of death 1999-2013 on cdc wonder online database, released 2015. https://www.cdc.gov/heartdisease/facts.htm.

[2] Virani S. S., Alonso A., Aparicio H. J. (2021). Heart disease and stroke statistics-2021 update: a report from the American Heart Association. *Circulation*.

[3] Tsao C. W., Aday A. W., Almarzooq Z. I., Beaton A. Z., Bittencourt M. S., Boehme A. K. (2023). Heart disease and stroke statistics—2023 update: a report from the american heart association. *Circulation*.

[4] Wang Z. W. (2023). Status of cardiovascular disease in China. *Journal of Geriatric Cardiology*.

[5] cardiovascular (2024). European alliance for cardiovascular health. https://www.cardiovascular-alliance.eu/cvd-facts-figures/.

[6] Choi H. E., Kim C., Lee D. J., Joo J. E., Kim H. S. (2023). Participation and prognostic impact of cardiac rehabilitation after acute coronary syndrome: big-data study of the Korean national health insurance Service. *Journal of Korean Medical Science*.

[7] Koenen R. R., Weber C. (2010). Platelet-derived chemokines in vascular remodeling and atherosclerosis. *Seminars in Thrombosis and hemostasis*.

[8] Investigators S., Benavente O. R., Hart R. G., McClure L. A., Szychowski J. M., Coffey C. S. (2012). Effects of clopidogrel added to aspirin in patients with recent lacunar stroke. *New England Journal of Medicine*.

[9] Ferguson A. D., Dokainish H., Lakkis N. (2008). Aspirin and clopidogrel response variability: review of the published literature. *Texas Heart Institute Journal*.

[10] Adam F., Kauskot A., Nurden P. (2010). Platelet JNK1 is involved in secretion and thrombus formation. *Blood*.

[11] Senis Y. A., Mazharian A., Mori J. (2014). Src family kinases: at the forefront of platelet activation. *Blood*.

[12] Singh P. A., Bajwa N., Chinnam S., Chandan A., Baldi A. (2022). An overview of some important deliberations to promote medicinal plants cultivation. *Journal of Applied Research on Medicinal and Aromatic Plants*.

[13] Wang T. H., Bhatt D. L., Topol E. J. (2006). Aspirin and clopidogrel resistance: an emerging clinical entity. *European Heart Journal*.

[14] Kim J. H. (2018). Pharmacological and medical applications of Panax ginseng and ginsenosides: a review for use in cardiovascular diseases. *Journal of ginseng research*.

[15] Irfan M., Kwak Y. S., Han C. K., Hyun S. H., Rhee M. H. (2020). Adaptogenic effects of Panax ginseng on modulation of cardiovascular functions. *Journal of Ginseng Research*.

[16] Wahab A., Sim H., Choi K. (2023). Antioxidant and anti-inflammatory activities of *Lespedeza cuneata* in Coal fly ash-induced murine alveolar macrophage cells. *Korean Journal of Veterinary Research*.

[17] Kim N., Kim J. (2015). A study on effects of *Lespedeza cuneata* extract on the improvement of scalp conditions in adult Men in Their 30∼ 40s. *Journal of the Korean Society of Cosmetology*.

[18] Park B. K., Kim C. W., Kwon J. E. (2019). Effects of *Lespedeza Cuneata* aqueous extract on testosterone-induced prostatic hyperplasia. *Pharmaceutical Biology*.

[19] Ha S. J., Lee J., Song K. M. (2018). Ultrasonicated *Lespedeza cuneata* extract prevents TNF-*α*-induced early atherosclerosis in vitro and in vivo. *Food & Function*.

[20] Cho E. J., Lee S. G., Kim D. O. (2009). The effect of *Lespedeza cuneata* extract for antioxidative and whitening effect. *International Journal of Life Science Research Archive*.

[21] Irfan M., Kwon H. W., Lee D. H. (2020). Ulmus parvifolia modulates platelet functions and inhibits thrombus formation by regulating integrin *α*IIb*β*3 and cAMP signaling. *Frontiers in Pharmacology*.

[22] Irfan M., Jeong D., Saba E. (2019). Gintonin modulates platelet function and inhibits thrombus formation via impaired glycoprotein VI signaling. *Platelets*.

[23] Tucker K. L., Sage T., Gibbins J. M. (2012). Clot retraction. *Platelets and Megakaryocytes: Volume 3, Additional Protocols and Perspectives*.

[24] Sachs U. J., Nieswandt B. (2007). In vivo thrombus formation in murine models. *Circulation Research*.

[25] Seong H. R., Wang C., Irfan M. (2022). DK-MGAR101, an extract of adventitious roots of mountain ginseng, improves blood circulation by inhibiting endothelial cell injury, platelet aggregation, and thrombus formation. *Journal of Ginseng Research*.

[26] Shim Y., Kwon I., Park Y. (2021). Characterization of ferric chloride-induced arterial thrombosis model of mice and the role of red blood cells in thrombosis acceleration. *Yonsei Medical Journal*.

[27] Chou J., Mackman N., Merrill-Skoloff G., Pedersen B., Furie B. C., Furie B. (2004). Hematopoietic cell-derived microparticle tissue factor contributes to fibrin formation during thrombus propagation. *Blood*.

[28] Steinhubl S. R., Moliterno D. J. (2005). The role of the platelet in the pathogenesis of atherothrombosis. *American Journal of Cardiovascular Drugs*.

[29] Pankov R., Yamada K. M. (2002). Fibronectin at a glance. *Journal of Cell Science*.

[30] Augustine T. N., van der Spuy W. J., Kaberry L. L., Shayi M. (2016). Thrombin-mediated platelet activation of lysed whole blood and platelet-rich plasma: a comparison between platelet activation markers and ultrastructural alterations. *Microscopy and Microanalysis*.

[31] Blair P., Flaumenhaft R. (2009). Platelet *α*-granules: basic biology and clinical correlates. *Blood Reviews*.

[32] Holmsen H. (1994). Significance of testing platelet functions in vitro. *European Journal of Clinical Investigation*.

[33] Park J. Y., Oh W. J., Kwak D. M. (2011). The anti-platelet activity of Hypsizygus marmoreus extract is involved in the suppression of intracellular calcium mobilization and integrin *α*IIb*β*3 activation. *Journal of Medicinal Plants Research*.

[34] Liao J. K., Seto M., Noma K. (2007). Rho kinase (ROCK) inhibitors. *Journal of Cardiovascular Pharmacology*.

[35] Bledzka K., Qin J., Plow E. F. (2019). Integrin alphaiibbeta: from discovery to efficacious therapeutic target. *Circulation research*.

[36] Adam F., Kauskot A., Rosa J., Bryckaert M. (2008). Mitogen‐activated protein kinases in hemostasis and thrombosis. *Journal of Thrombosis and Haemostasis*.

[37] Liu G., Yuan Z., Tian X. (2021). Pimpinellin inhibits collagen-induced platelet aggregation and activation through inhibiting granule secretion and PI3K/Akt pathway. *Frontiers in Pharmacology*.

[38] L’Abbate S., Nicolini G., Forini F. (2020). Myo–inositol and D-Chiro–inositol oral supplementation ameliorate cardiac dysfunction and remodeling in a mouse model of diet-induced obesity. *Pharmacological Research*.

[39] Schantl A. E., Verhulst A., Neven E. (2020). Inhibition of vascular calcification by inositol phosphates derivatized with ethylene glycol oligomers. *Nature Communications*.

[40] Jing L., Yanyan Z., Junfeng F. (2015). Acetic acid in aged vinegar affects molecular targets for thrombus disease management. *Food & Function*.

[41] Gambert S., Héliès-Toussaint C., Grynberg A. (2007). Extracellular glycerol regulates the cardiac energy balance in a working rat heart model. *American Journal of Physiology- Heart and Circulatory Physiology*.

[42] Tayyeb J. Z., Popeijus H. E., Mensink R. P., Konings M. C., Mokhtar F. B., Plat J. (2020). Short-chain fatty acids (except hexanoic acid) lower NF-kB transactivation, which rescues inflammation-induced decreased apolipoprotein AI transcription in HepG2 cells. *International Journal of Molecular Sciences*.

[43] Chu X., Zhang J., Li Y. (2023). Dimethyl fumarate possesses antiplatelet and antithrombotic properties. *International Immunopharmacology*.

[44] Motoyama K., Nagata T., Kobayashi J. (2018). Discovery of a bicyclo [4.3. 0] nonane derivative DS88790512 as a potent, selective, and orally bioavailable blocker of transient receptor potential canonical 6 (TRPC6). *Bioorganic & Medicinal Chemistry Letters*.

[45] Ren K., Duan W., Liang Z. (2020). Glutaraldehyde and 2, 3-butanediol treatment of bovine pericardium for aortic valve bioprosthesis in sheep: a preliminary study. *Annals of Translational Medicine*.

