# Supplementary material for: Antiplatelet and Antithrombotic Activities of Lespedeza cuneata via Pharmacological Inhibition of Integrin αIIbβ3, MAPK, and PI3K/AKT Pathways and FeCl3-Induced Murine Thrombosis
Source: Evid Based Complement Alternat Med. 2024 Feb 9;2024:9927160. doi: 10.1155/2024/9927160 (PMC10872769; doi:10.1155/2024/9927160)

**Supplementary Material**

**Figure legends:** Various quantities of *L. cuneata* extracts with 1 mM CaCl_2_ were preincubated using washed platelets for 1 min at 37°C before collagen stimulation for 5 min with constant stirring. By adding a lysis buffer, platelet aggregation stopped, and the protein concentration was calculated using the BCS assay (PRO-MEASURE; iNtRON Biotechnology). In a 10% SDS-PAGE, total platelet proteins were isolated, and then they were transferred to PVDF membranes. Membranes were blocked with 5% skim milk, probed with the appropriate antibodies (phospho-ERK, phospho-JNK, phospho-p38MAPK, phospho-PI3K, phospho-Akt, etc.), and then observed using enhanced chemiluminescence.

The full-length blots for the gel images are given below in the pictures.

Supplementary figure S1a= P-ERK pathway in platelets

Supplementary figure S1b= P-p38 pathway in platelets

Supplementary figure S1c= P-JNK pathway in platelets

Supplementary figure S1d= P-Akt pathway in platelets

Supplementary figure S1e= PI3K pathway in platelets

**Western blot bands**

**S1a**

150-

130-

100-

70-

53-

41-

30-

22-

**
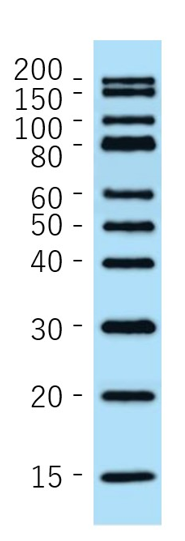
**

Resting collagen 50 100 200 PD

Resting collagen 50 100 200 PD

**
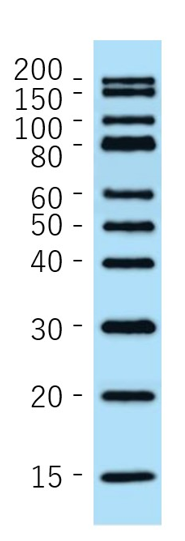
**

Resting collagen 50 100 200 PD

Resting collagen 50 100 200 PD

**
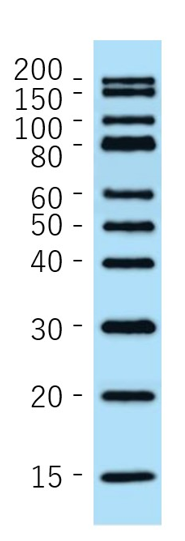
**

Resting collagen 50 100 200 PD

Resting collagen 50 100 200 PD

**
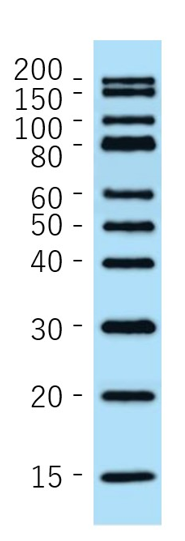
**

**
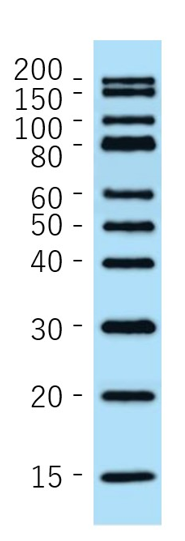
**

**P-ERK (42-44 kDa) T-ERK**


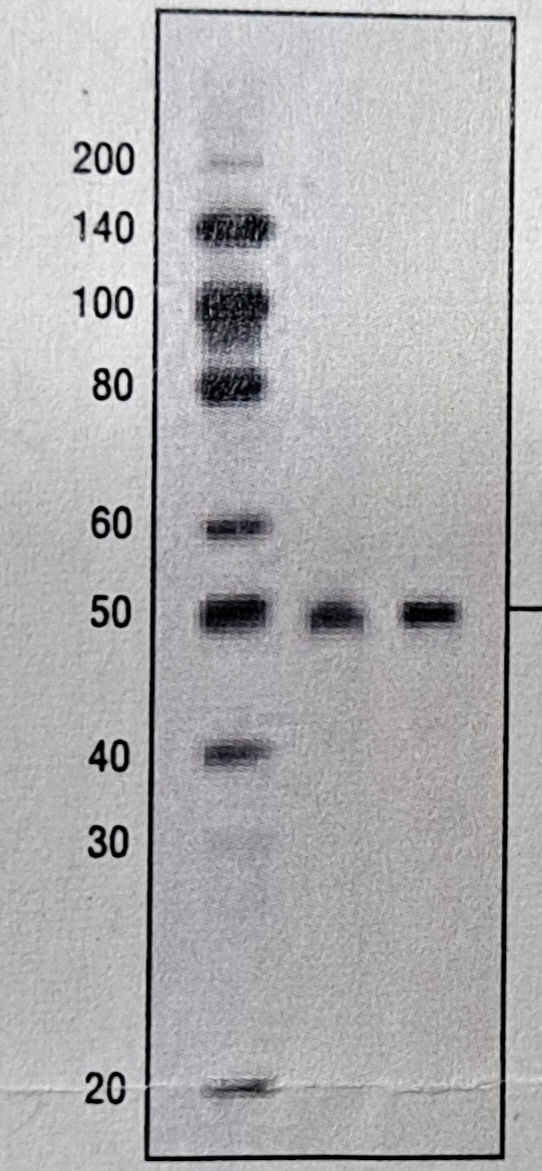

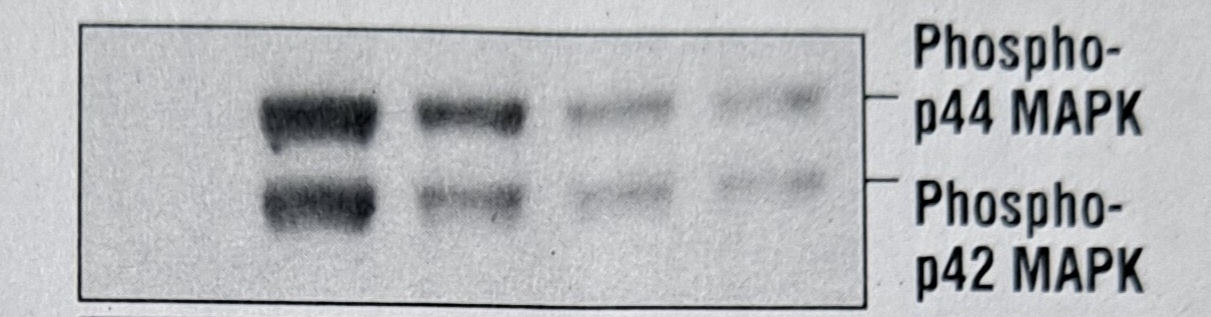


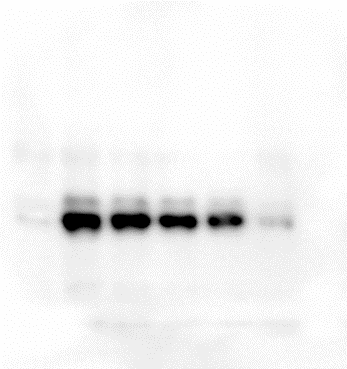

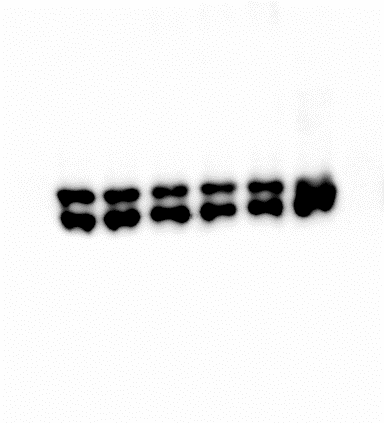


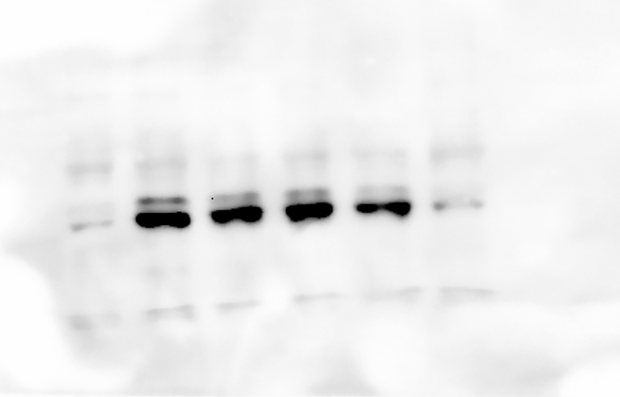

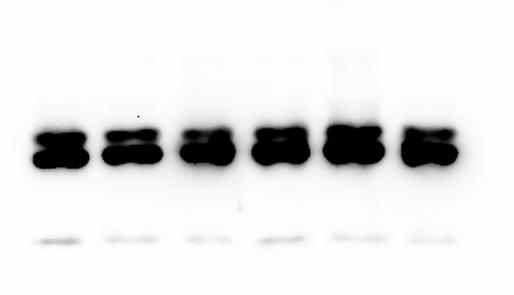


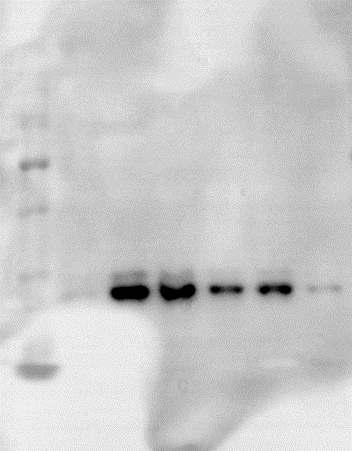

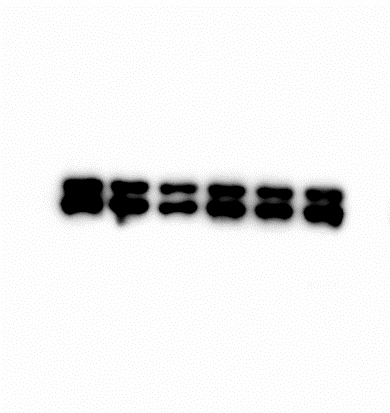


**S1b**

150-

100-

70-

53-

41-

30-

150-

100-

70-

53-

41-

30-

Resting collagen 50 100 200 SB

**
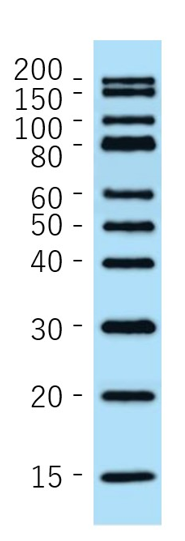
**

Resting collagen 50 100 200 SB

**
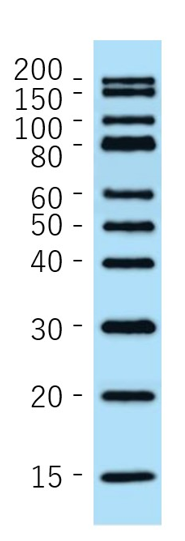
**

Resting collagen 50 100 200 SB

**
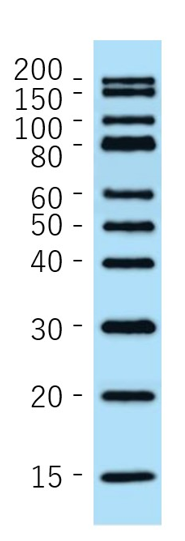
**

Resting collagen 50 100 200 SB

**
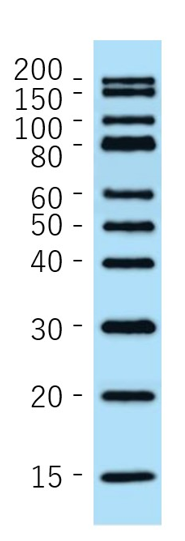
**

Resting collagen 50 100 200 SB

Resting collagen 50 100 200 SB

**P-p38 (44 kDa) T-p38**


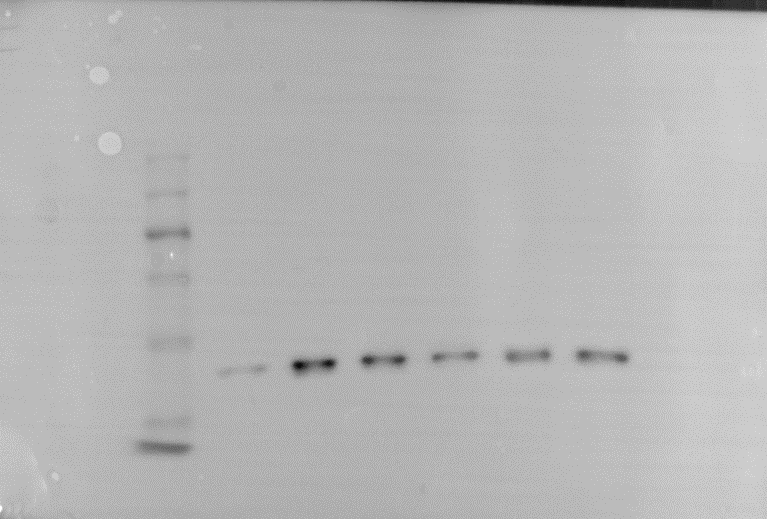

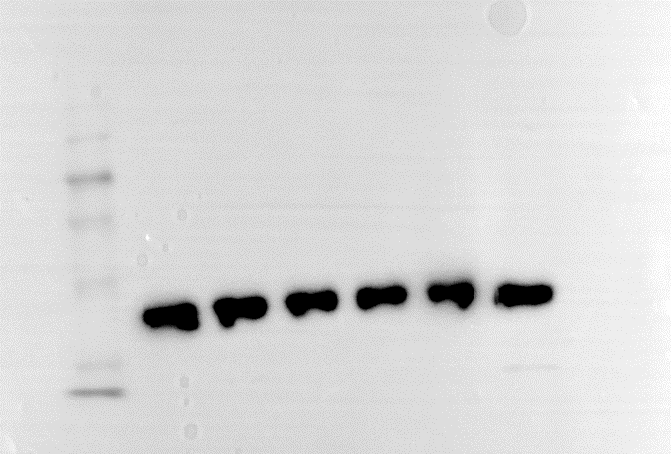


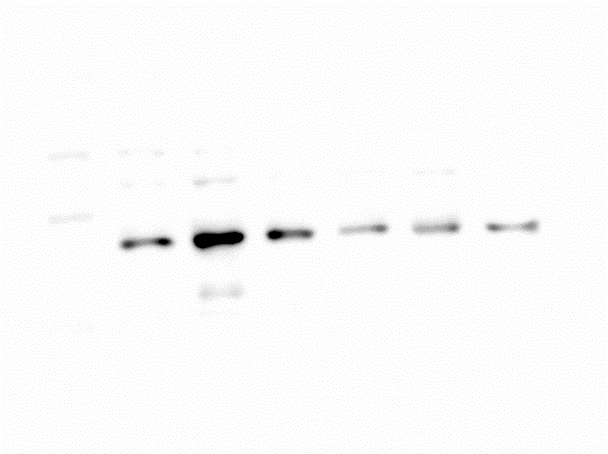

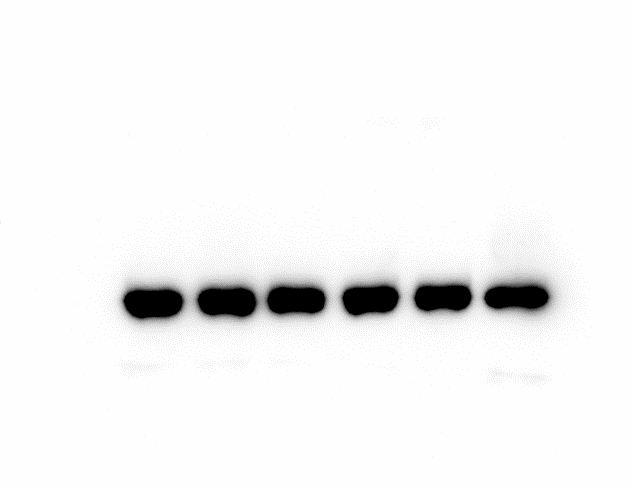


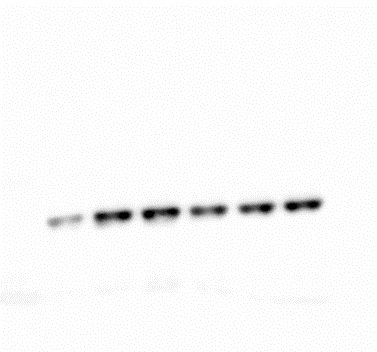

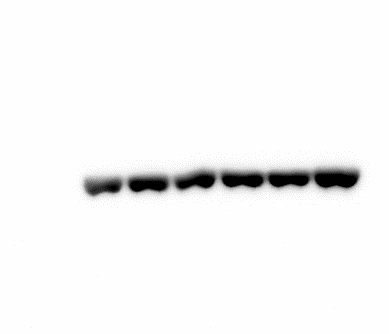


**S1c**

Resting collagen 50 100 200 SP

**
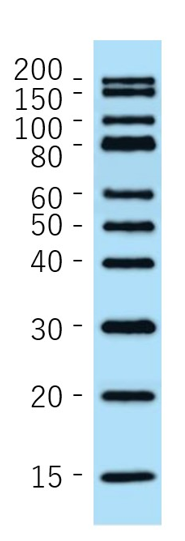
**

Resting collagen 50 100 200 SP

**
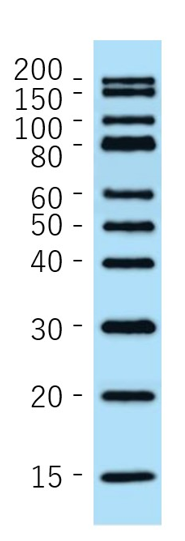
**

Resting collagen 50 100 200 SP

**
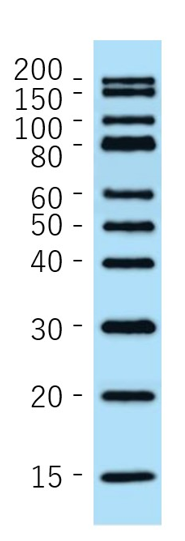
**

Resting collagen 50 100 200 SP

**
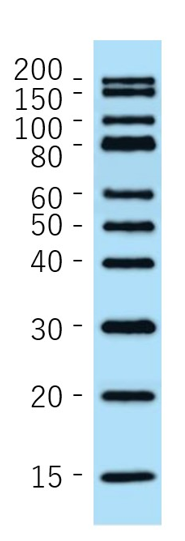
**

**
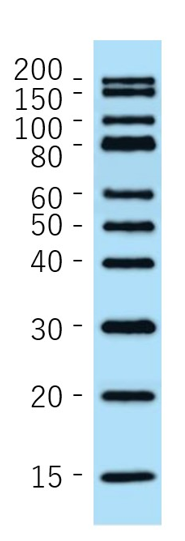
**

**
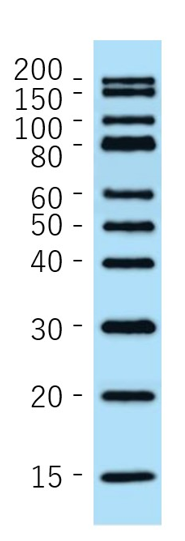
**

Resting collagen 50 100 200 SP

Resting collagen 50 100 200 SP

**P-JNK (46-54 kDa) T-JNK**


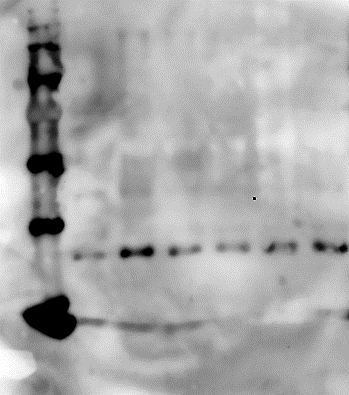

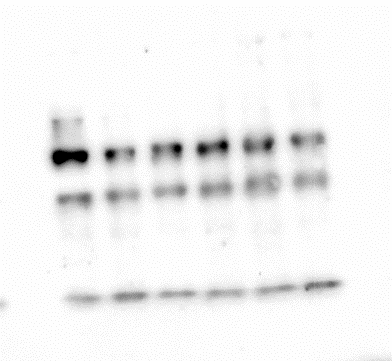


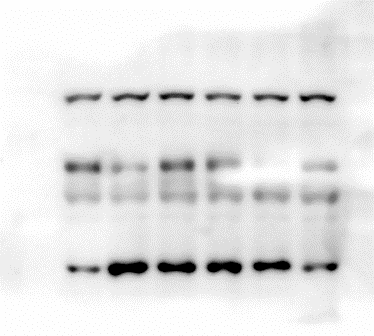

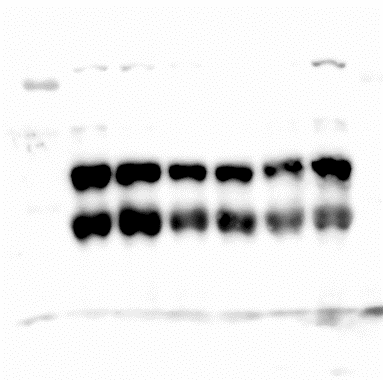


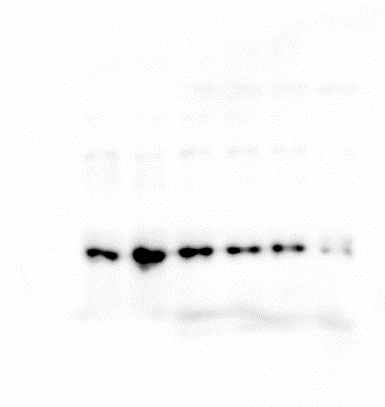

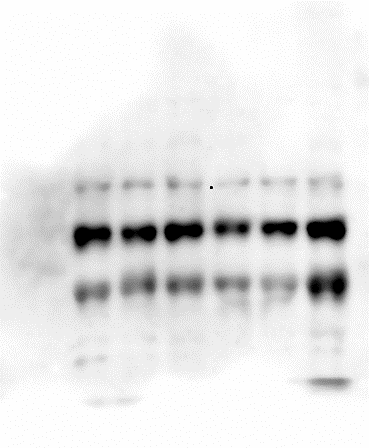


**S1d**


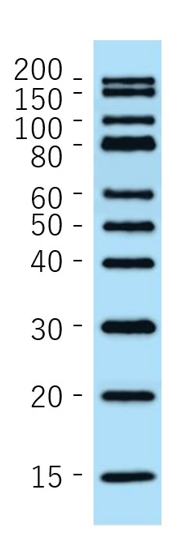


Resting collagen 50 100 200 LY

Resting collagen 50 100 200 LY


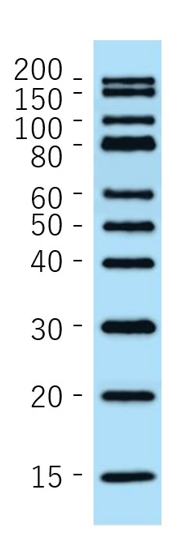


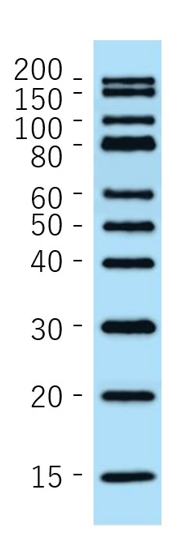


Resting collagen 50 100 200 LY

Resting collagen 50 100 200 LY


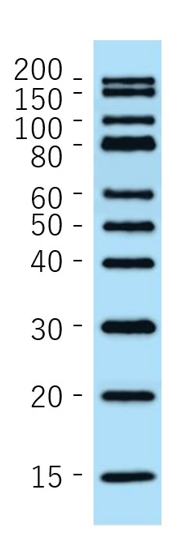


Resting collagen 50 100 200 LY


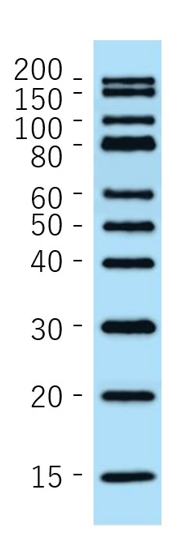


Resting collagen 50 100 200 LY


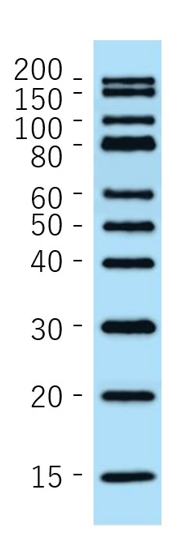


**P-Akt (60 kDa) T-Akt**


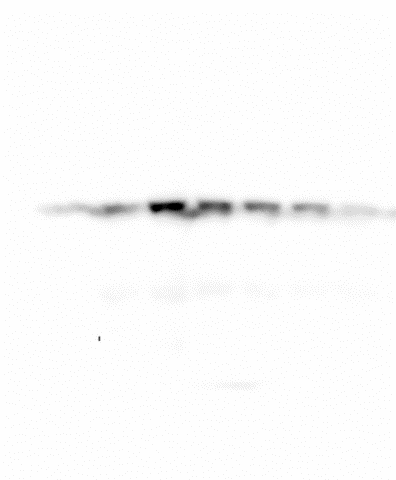

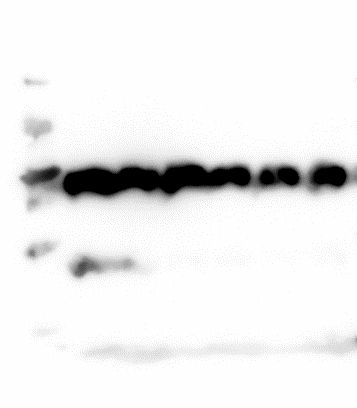


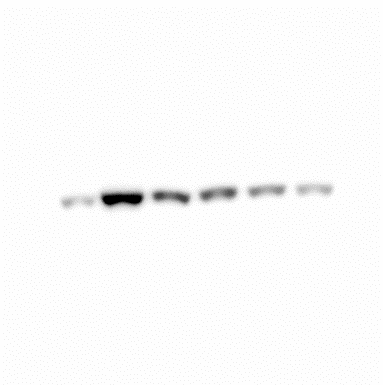

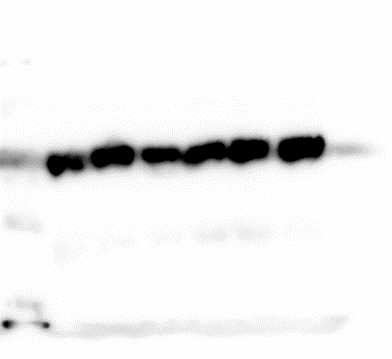


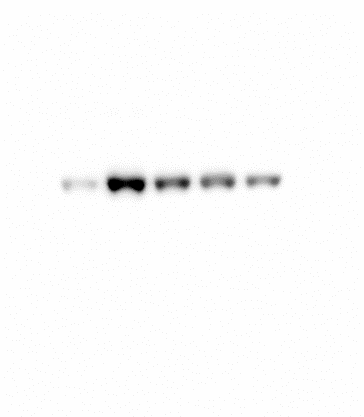

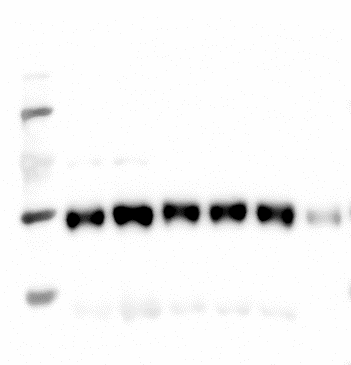


**S1e**

Resting collagen 50 100 200 LY

Resting collagen 50 100 200 LY

Resting collagen 50 100 200 LY

Resting collagen 50 100 200 LY

Resting collagen 50 100 200 LY

Resting collagen 50 100 200 LY


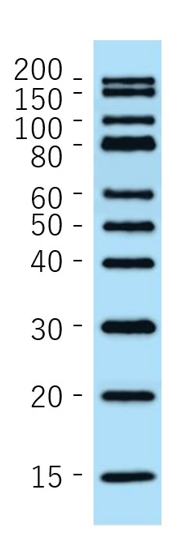


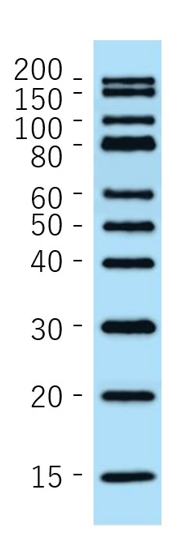


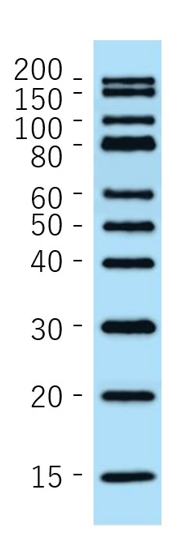


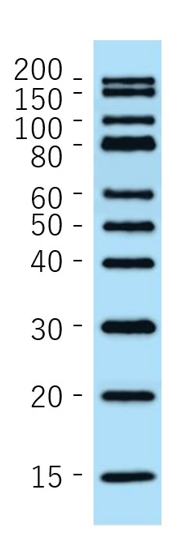


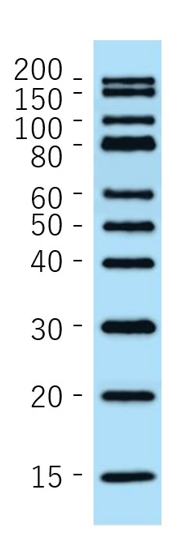


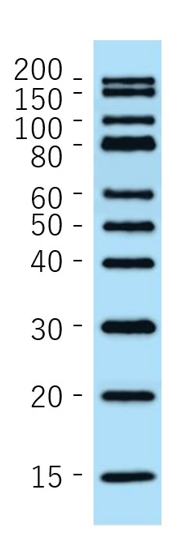


**P-PI3K (85 kDa) T-PI3K**


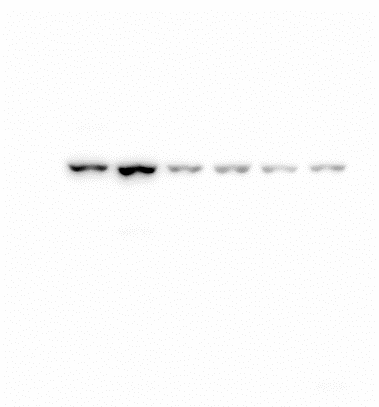

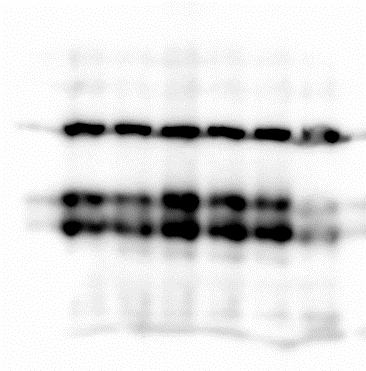


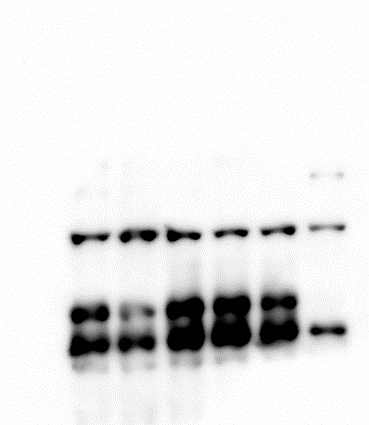

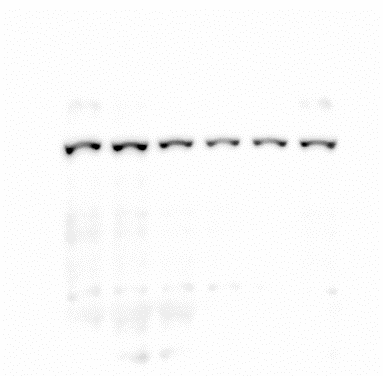


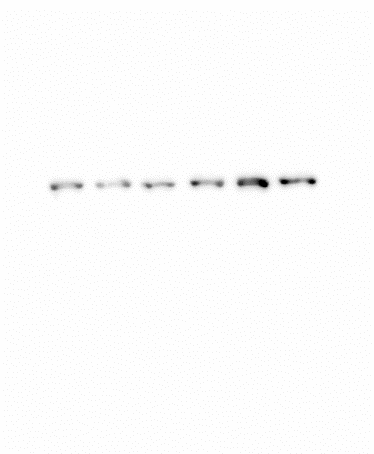

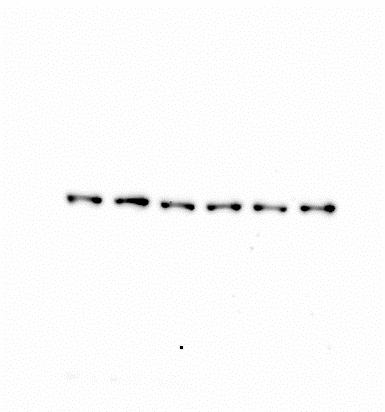

Supplement: Supplementary Materials — The full-length blots for the gel images are shown in the supplementary material. Supplementary figure S1a = P-ERK pathway in platelets. Supplementary figure S1b = P-p38 pathway in platelets. Supplementary figure S1c = P-JNK pathway in platelets. Supplementary figure S1d = P-Akt pathway in platelets. Supplementary figure S1e = PI3K pathway in platelets. [file 9927160.f1.docx]
